# Forensic application of comet assay: an emerging technique

**DOI:** 10.1080/20961790.2017.1379893

**Published:** 2017-10-09

**Authors:** Ritesh Kumar Shukla

**Affiliations:** Division of Biological and Life Sciences, School of Arts and Sciences, Ahmedabad University, Ahmedabad, India

**Keywords:** Forensic science, comet assay, PMI, single-cell gel electrophoresis, DNA fragmentation, DNA repair, forensic application

## Abstract

Postmortem interval (PMI) estimation is a recurring problem in the field of forensic medicine. Conventional methods are effective but are insufficient to estimate accurate and precise time of death or PMI. In addition, degradation of biological samples is another major problem in forensic science which affects the investigation process and misleads the result. Some previous studies reported that DNA fragmentation has strong correlation with PMI. DNA fragmentation increased with prolonged PMI. Comet assay is a rapid sensitive, versatile, reliable and cost effective technique that is specifically used for qualitative and quantitative estimation of nuclear DNA fragmentation. Due to this attribute, comet assay can help to estimate accurate and precise time of death for some extent that is for early PMI estimation. In addition, two confounding factors are responsible for DNA fragmentation: (1) micro-organism; (2) environmental condition. Here, comet assay plays a dual role: (1) partially degraded samples get repaired using repair enzyme; (2) accurate time since deposition can be measured without using repair enzyme. Furthermore, this assay can also help to identify potential exposures of environmental-released chemicals/toxicants and its deleterious effects on human population. In this way, comet assay shows its versatile applications that could be useful for forensic investigation. Therefore, with the help of this review, an attempt was made to explore the versatility of comet assay technique for forensic applications and its future perspective.

## Introduction

Forensic science is a well-versed branch of science which applied the principles and methods of basic sciences in the investigation and helps to establish the authenticity of facts or evidence in a court of law. In this sequence, many well-developed methods and techniques used in molecular biology and toxicology can also help in forensic investigations. These methods and techniques can permit the sensitive detection/quantification of DNA from biological fluids like blood, semen and saliva. Some of these techniques are polymerase chain reaction (PCR), enzyme linked immunosorbent assay (ELISA), flow cytometer, agarose gel electrophoresis and immunoelectrophoresis [1–5]. In this sequence, single-cell gel electrophoresis (SCGE) assay is a rapid sensitive versatile and cost effective technique that is specifically used for qualitative and quantitative estimation of nuclear DNA fragmentation [6]. This technique is mainly specific and sensitive in detecting single- and double-stranded breaks of DNA, alkali-labile and excision repair sites in individual cells [6,7]. This technique is applied specifically to almost any type of eukaryotic cells that can be obtained as a single-cell suspension. This assay requires very small number of cell samples (minimum 10 000 cells) and results can be obtained in a single day. This technique is sensitive to detect DNA damage in viable cells only. Once cells are dead, it is unable to quantify DNA damage precisely [7]. Additionally, performing cost of this assay is judiciously cost effective.

**Figure 1.**
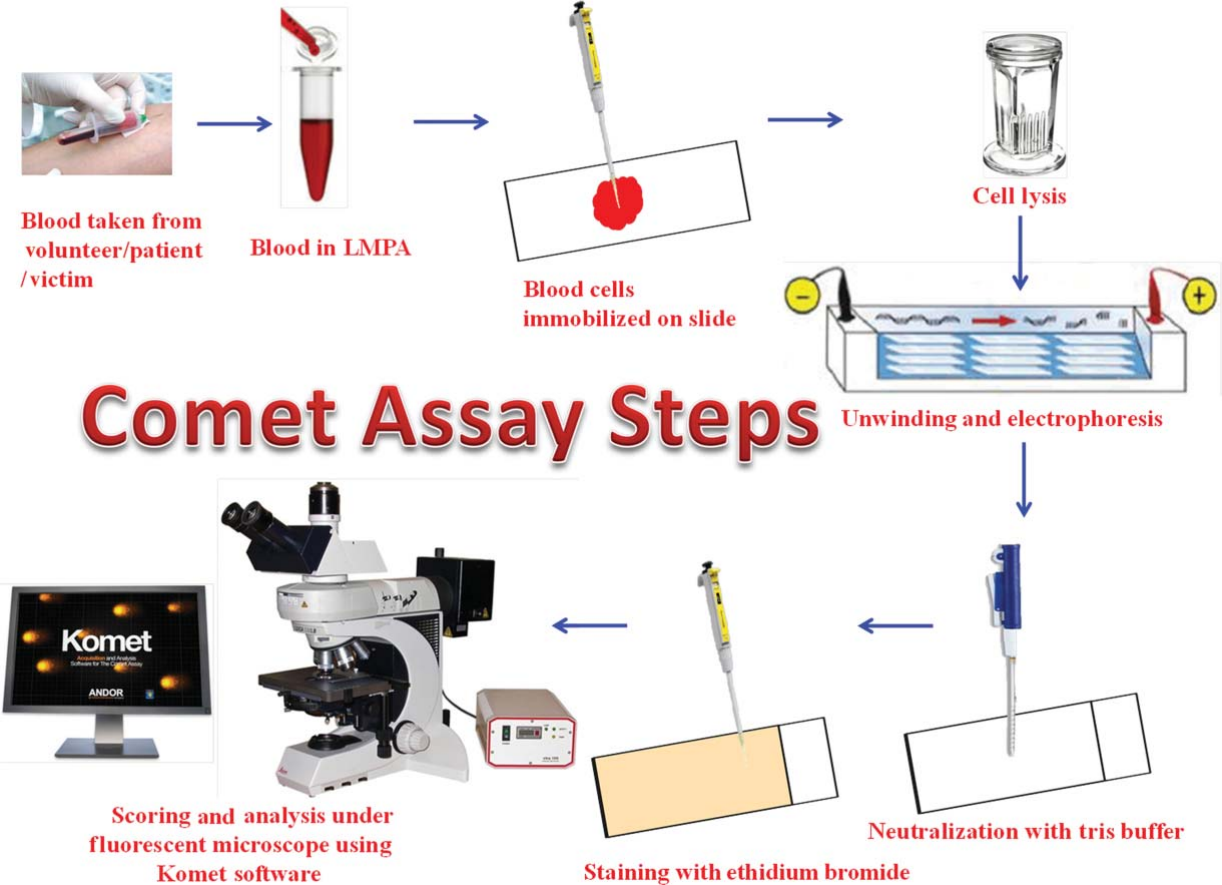
A schematic representation of comet assay steps.

The principle of this technique is based upon migration of damaged/degraded DNA. Damaged DNA molecules can migrate more readily in an electric field compared to intact molecules [7]. In this technique (Figure 1), biological sample was first diluted in phosphate buffered saline and prepared as single-cell suspension. This single-cell suspension was then mixed with low melting agarose (LMA) gel and placed on normal agarose gel pre-coated microscope slide. Subsequently, a cover slip is placed gently to evenly spread the cells in the agarose. After the gel solidified, again cover slip removed gently and a third layer of LMA was added onto the slide to prevent uneven migration of DNA in the two layers. Again cover slip is placed over third layer of LMA and kept the slide on ice pack to solidify the gel. Finally, when the gel solidified onto the slide, the cover slips are removed and slides immersed in lysing solution to remove proteins, smaller DNA molecules that can be able to migrate away from the residual nucleus. After lysis, unwinding, electrophoresis, neutralization and stained with fluorescent DNA binding dye are the following steps before microscopic analysis of the slides. Slides are then scored using “Komet”, an image analysis software (Kinetic Imaging, Andor Technology, Belfast, UK) attached to a fluorescence microscope (Leica, Germany) equipped with appropriate filters (N2.1, excitation wavelength of 515–560 nm and emission wavelength of 590 nm).

**Figure 2.**
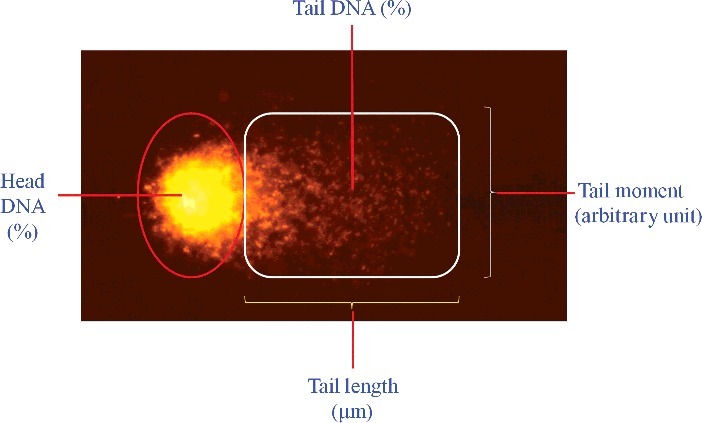
Photomicrograph showing comet parameters for DNA fragmentation/damage assessment. Tail moment = Tail length × Tail DNA (%)

The observed image appears as comets with a head region containing intact DNA and a tail containing fragmented DNA. The parameters used to assess DNA fragmentation in the cells are tail length (μm), tail DNA (%) and tail moment (Figure 2).

This technique, because of its simplicity, sensitivity and small number of sample requirement, would become an ideal detection system for forensic applications. With the help of this review, an attempt was made to insight the versatility of SCGE technique for forensic applications and its future perspective.

## Forensic application of comet assay

### Estimation of postmortem interval using SCGE

Accurate postmortem interval (PMI) estimation is a recurring problem in the field of forensic medicine. In most of the homicide cases, dead body was apprehended within the first 48 h. In this time frame, quick, accurate and precise PMI estimation is a critical task [8]. At present, various methods of PMI estimation have been used, but none can provide accurate and precise estimation of PMI better than 8 h window [9].

To circumvent this problem, Johnson and Ferris [9] introduced SCGE technique to estimate PMI with the help of qualitative and quantitative assessment of DNA fragmentation. It is well known that upon the death of an organism, nucleases within the cells cause DNA fragmentation which increases with time. The results of this study revealed that with an increased PMI from 0 to 56 h, DNA fragmentation was also increased which is evident by comet assay parameters, namely olive tail moment (OTM), tail length and tail DNA. These parameters provide strong statistical positive correlation with PMI using linear regression correlation method.

Similar findings also observed by Lin et al. [10] reported that rate of DNA degradation of rat liver cells had a linear correlation with early postmortem period. However, Chen et al. [11] also exhibited that amount of DNA from heart, liver and kidney cells had rapidly degraded in the first 6 h after death which indicates a linear correlation between DNA fragmentation and PMI.

These findings coincide with Luo et al. [12] and Zhen et al. [13] who stated that DNA fragmentation as evident by comet tail increased with prolongation of PMI. In addition, Hao et al. [14] observed that DNA fragmentation induced with time-dependent manner using comet assay at different PMI (0, 3, 6, 9, 12 and 24 h) in the brain and liver cells of rats. Similar results also observed by Gomaa et al. [15] which reported that DNA fragmentation increased with prolongation of PMI (3–24 h) in brain and liver samples.

Furthermore, Zheng et al. [16] studied on DNA fragmentation in mouse brain and dental pulp cells using comet assay at prolonged PMI up to 72 h. This study revealed a high correlation between comet parameters and PMI through linear regression analysis.

These findings recommended that SCGE is a more sensitive, reliable and expedient technique that could be used as a supportive method along with other conventional methods for PMI estimation (Table 1).

**Table 1.** Correlation between DNA degradation and postmortem interval (PMI) at different time points.

Study	Species	Time frame assessed	Remarks
[10]	Liver cells of rats	0–24 h	Linear correlation between DNA degradation and early PMI in liver cells.
[9]	Liver cells	0–56 h	DNA degradation detected up to 24 h in liver cells.
[11]	Corpse (human)	6–48 h	DNA of heart, liver and kidney of human had a rapid degraded in first 6 h after death. Good correlation between DNA degradation of spleen cells and PMI.
[12]	Bone marrow	Up to 14 days	Gradual degradation of bone marrow DNA with extension of PMI.
[13]	Myocardium cells of mice	0–72 h	DNA degradation of myocardium cells has a linear correlation with PMI up to 72 h.
[14]	Brain and liver cells of rats	0, 3, 6,9, 12 and 24 h	Estimation of nucleic acids degradation is well-versed alternative to classical methods for PMI estimation.
[8]	Brain, lungs, spleen, liver and skeletal muscles of drowned rats	0, 3, 6, 12 and 24 h	Linear relationship between the degradation rate of nuclear DNA and PMI in liver cells. Brain shows slower rate of DNA degradation, so can be used for longer PMI estimation.
[16]	Mouse brain and dental pulp cells	0–72 h	DNA degradation of brain and dental pulp cells have high correlation with extended PMI.
[15]	Brain and liver cells of albino rats	3–24 h	DNA degradation in brain and liver cells increased with PMI.

### Time since deposition estimation using SCGE

Time since deposition estimation of a biological stain could be a valuable evidence for forensic investigation. It helps to scrutinize at what time this stain has been left at the crime scene which consequently aids to determine at what time the crime has been occurred.

In this sequence, time since estimation of a rape/sexual assault case has been a persistent problem [17]. In sexual assault cases, dried seminal fluid, which contains spermatozoa, is usually found on victim's cloth. These spermatozoa contain DNA and its fragmentation could be helpful to correlate it with time since deposition at the crime scene/cloth of victim [9]. In addition, the idea of DNA fragmentation correlated with PMI estimation is now new in forensic investigation. With this hypothesis, Miteva et al. [17] have shown the fragmentation pattern of sperm DNA using comet assay at different time points of deposition. The results revealed that fragmentation of sperm DNA induced with prolonged time since deposition of stain as evident by comet assay parameter. These comet assay parameters, i.e. OTM and tail length, have been increased with time-dependent manner. This study concluded that DNA fragmentation in the spermatozoa could be a “molecular clock” for crime investigation.

### Environmental degradation assessment of biological fluids using SCGE

Environmental damage to DNA can mainly initiate from micro-organisms and atmospheric conditions [18,19]. Both of these contributors/factors are influenced by the geographical location and local environment of the sample. It is believed that environmental conditions are mainly responsible for degradation of biological fluids recovered from the scene of crime [19]. But it is still not clear which type of factor is more responsible. Environmentally degraded biological fluids may result as the loss of signal in short tandem repeat (STR) profiling that affects the genotyping which becomes problematic for investigator during result interpretation.

In a previous study, Lehmann et al. [20] stated that damaged DNA contains nucleotide modifications in the DNA strands which block the normal DNA replication machinery of the cell. As a consequence, damaged DNA segments cannot be amplified by PCR and it enhances the chances of misleading results. DNA can be damaged in a number of ways resulting in breaks in the strands or removal or chemical alteration of the nucleotide bases. The extent of DNA fragmentation can vary, but if it persists for longer period without being repaired, then probability of DNA amplification and analysis will decline [19].

Ballantyne [18] demonstrated that DNA damage encountered in forensically relevant stains occurs due to environmental exposure (exogenous, UV irradiation, heat, humidity and micro-organism growth). DNA damage can be varied such as strand breaks (single and double-stranded breaks), base modifications and to a lesser extent, DNA–DNA crosslinks [21,22]. In this study, three repair systems (direct reversal by photolyase and single-stranded break/gap repair) were developed to repair various types of damages. The single-stranded break/gap repair system has proven successful in the recovery of a genetic signature from both laboratory-damaged and environmentally exposed bloodstains. Furthermore, in another study, Nelson [23] reported that DNA is double stranded and thus redundantly structured. This redundancy in structure has created a rich collection of repair mechanisms for different kinds of damage, often using the information in one strand to reconstruct the other. Findings from the study revealed that biological samples exposed to conditions such as radiation, alkylation and fenton reaction can modify the chemical structure of the DNA which may concurrently induce unrepairable fragmentation in DNA strands. Moreover, when they introduced additional method of DNA repair using commercially available enzymes, partially fragmented DNA samples get repaired.

### Environmental forensics using SCGE

Environmental forensics generally involves the reconstruction of past environmental events, such as the timing, types and amounts, and sources of chemical releases to the environment [24]. These environmental-released chemicals directly or indirectly induce tissue- and cell-type specific DNA damage in exposed population [25]. Therefore, the technique dealing with individual cells seems to be an optimal one for environmental forensic investigation. In this prerogative, comet assay would be an ideal technique for assessing the genotoxic potential of environmental-released chemicals in sentinel organisms [26]. In a previous study, the extent of DNA damage in coelomocytes collected from earthworms found in different soil samples as an indicator of soil pollution was assessed by the comet assay [27]. Furthermore, the comet assay was also used to assess the extent of DNA damage in the population exposed to pesticides and other environmental pollutants [28]. Human bio-monitoring studies through the comet assay are not only generating data but also help to identify potential exposures of environmental-released chemicals/toxicants in human population which assist to predict disease risk [29,30]. These data will provide information to the environmental protection agencies to identify the hazardous chemical, its lethal concentration, release location and exposed population and type of disease [29–32]. This information would be beneficial for environmental forensic perspectives during investigation process. In this way, the comet assay is directly or indirectly associated with environmental forensic investigation process.

## Future perspective

Toxicovigilance refers to the continuous monitoring of toxic exposure of substances in the exposed population. It includes detection, identification, validation and its adverse consequences on the exposed population. The main purpose of toxicovigilance is preventing toxicological accidents rather than having to cure them. Toxicovigilance becomes the part of forensic when intentionally prolonged release of substance has shown its adverse effect on the population present in surroundings. In this case, SCGE could be used as screening method to assess the extent of DNA fragmentation in the population exposed to release toxic substance. For example, population residing near to lead industry may have highest probability to expose with lead residues. In addition, it is well known that exposure of lead caused DNA fragmentation. So in this type of industrial area, through the toxicovigilance process using SCGE assay, extent of DNA damage can be assessed in the exposed population.

## Conclusion

The future of DNA forensic will have an impact on other areas of forensic science. DNA plays a key for biological identification of a person which is an end point of forensic investigation. Degradation of biological samples is a very common problem in forensic science which adversely affects the result interpretation in the court. Results obtained from degraded sample will always be questionable in the court. Here, SCGE will play a key role and used as a preventive tool and screening method. Prior to STR profiling, degradation of biological fluids can be assessed through this assay as a screening method. In addition, with the help of this assay, not only DNA fragmentation can be assessed but its repair process could also be possible via introducing oxidative DNA repair enzyme like formamidopyrimidine-DNA glycosylase (FPG), Endo III, as a preventive tool.

## References

[1] Morling N PCR in forensic genetics. Biochem Soc Trans. 2009;37:438–440. 19290877 10.1042/BST0370438

[2] Cordeiro C, Seoane R, Camba A, et al. High variation in hypoxanthine determination after analytical treatment of vitreous humor samples. J Foren Sci. 2015;60:1346–1349. 10.1007/s12024-014-9590-3 25119241

[3] Shukla RK Forensic biotechnology: application of flow cytometry in legal medicine. Int J Foren Sci. 2016;1:1–2

[4] Hurley IP, Cook R, Laughton CW, et al. Detection of human blood by immunoassay for applications in forensic analysis. Foren Sci Int. 2009;190:91–97. 10.1016/j.forsciint.2009.05.018 19576708

[5] Perrigo BJ, Joynt BP Use of ELISA for the detection of common drugs of abuse in forensic whole blood samples. Can Soc Foren Sci J. 2013;28:261–269.

[6] Shukla RK, Bajpayee M, Dhawan A Detection of DNA damage in different organs of the mouse. Comet Assay Toxicol. 2016;30:164–176.

[7] Olive PL, Banath JP, Fjell CD DNA strand breakage and DNA structure influence staining with propidium iodide using the alkaline comet assay. Cytometry. 1994;16:305–312. 7527314 10.1002/cyto.990160404

[8] El-Harouny M, El-Dakroory S, Attalla S, et al. The relationship between postmortem interval and DNA degradation in different tissues of drowned rats. Internet J Foren Sci. 2008;4:1–7.

[9] Johnson LA, Ferris JAJ Analysis of postmortem DNA degradation by single-cell gel electrophoresis. Foren Sci Int. 2002;126:43–47. 10.1016/s0379-0738(02)00027-0 11955831

[10] Lin LQ, Liu L, Deng WN, et al. An experimental study on the relationship between the estimation of early post-mortem interval and DNA content of liver cells in rats by image analysis. Fa Yi Xue Za Zhi. 2000;16:68–69. 12536450

[11] Chen X, Shen YW, Gu YJ The research of relationship between DNA degradation and post-mortem interval. Fa Yi Xue Za Zhi. 2005;21:115–117. 15931752

[12] Luo GH, Chen YC, Cheng JD, et al. Relationship between DNA degeneration and postmortem interval of corrupt corpse. Fa Yi Xue Za Zhi. 2006;22:7–9. 16524174

[13] Zhen JL, Zhang XD, Niu QS Relationship between the postmortem interval and nuclear DNA changes of heart muscular cells in mice. Fa Yi Xue Za Zhi. 2006;22:173–176. 16856335

[14] Hao LG, Deng SX, Zhao XC Recent advancement in relationship between DNA degradation and postmortem interval. Fa Yi Xue Za Zhi. 2007;23:145–147. 17619465

[15] Gomaa Mie S, El-Khalek AMA, Sameer MM The relationship between the postmortem interval and the DNA degradation in brain and liver of adult albino rats. J Am Sci. 2013;9:535–540.

[16] Zheng J, Li X, Shan D, et al. DNA degradation within mouse brain and dental pulp cells 72 h post-mortem. Neural Regen Res. 2012;7:290–294. 25806071 10.3969/j.issn.1673-5374.2012.04.009 PMC4353102

[17] Miteva R, Georgieva M, Peycheva E, et al. Development of conditions for comet assay application in forensic investigation of rape and other sexual assaults. Biotechnol Biotech Equip. 2009;23:1093–1094.

[18] Ballantyne J. Assessment and In Vitro repair of damaged DNA templates. Forensic DNA research and development final report. National Institute of Justice; U.S. Department of Justice; 2006 p. 1–94. Available from: https://www.ncjrs.gov/pdffiles1/nij/grants/214166.pdf

[19] Goodwin CS In vitro repair of gamma-irradiated DNA for forensic analysis [thesis]. University of Canberra; 2013 Available from: http://www.canberra.edu.au/researchrepository/file/ff8d464f-9eb5-4cdba069-40f05eae8b00/1/full_text.pdf

[20] Lehmann AR. Replication of damaged DNA. Cell Cycle. 2003;2:300–302. 12851478

[21] Hall A, Ballantyne J Characterization of UVC-induced DNA damage in bloodstains: forensic implications. Anal Bioanal Chem. 2004;380:72–83. 15309360 10.1007/s00216-004-2681-3

[22] Hall A, Sims LM, Ballantyne J Assessment of DNA damage induced by terrestrial UV irradiation of dried bloodstains forensic implications. Foren Sci Int Genet. 2014;8:24–32. 10.1016/j.fsigen.2013.06.010 24315585

[23] Nelson J. Repair of damaged DNA for forensic analysis. U.S. Department of Justice; 2009 Available from: https://www.ncjrs.gov/pdffiles1/nij/grants/227498.pdf

[24] Philp RP An overview of environmental forensics. Geol Acta. 2014;12:363–374.

[25] De Lapuente J, Lourenço J, Mendo SA, et al. The comet assay and its applications in the field of ecotoxicology: a mature tool that continues to expand its perspectives. Front Genet. 2015;6:1–20. 26089833 10.3389/fgene.2015.00180 PMC4454841

[26] Tice RR The single cell gel/comet assay. In: Philips DH, Venitt S, editors. Environmental mutagenesis. Oxford: BIOS Scientific Publishers; 1995 p. 315–339.

[27] Verschaeve L, Gilles J, Schoctors J, et al. The single cell gel electrophoresis technique or comet test for monitoring dioxin pollution and effects. In: Fiedler H, Frank H, Hutzinger O, Parzefall W, Riss A, Safe S, editors. Organohalogen compounds 11. Vienna: Federal Environmental Agency; 1993 p. 213–216.

[28] Błasiak J, Trzeciak A Single cell gel electrophoresis (comet assay) as a tool for environmental biomonitoring. An example of pesticides. Pol J Environ Stud. 1998;7:189–194.

[29] Anderson D, Dhawan A, Laubenthal J The comet assay in human biomonitoring. Methods Mol Biol. 2013;1044:347–362. 23896886 10.1007/978-1-62703-529-3_18

[30] Guidelines for the health risk assessment of chemical mixtures. Washington (DC): U.S. Environmental Protection Agency; 1986.

[31] Supplementary guidance for conducting health risk assessment of chemical mixtures. Washington (DC): U.S. Environmental Protection Agency; 2000.

[32] US-EPA framework for cumulative risk assessment. Washington (DC): U.S. Environmental Protection Agency; 2003.

